# An attenuated strain of *Bacillus anthracis *(CDC 684) has a large chromosomal inversion and altered growth kinetics

**DOI:** 10.1186/1471-2164-12-477

**Published:** 2011-09-30

**Authors:** Richard T Okinaka, Erin P Price, Spenser R Wolken, Jeffrey M Gruendike, Wai Kwan Chung, Talima Pearson, Gary Xie, Chris Munk, Karen K Hill, Jean Challacombe, Bruce E Ivins, James M Schupp, Stephen M Beckstrom-Sternberg, Arthur Friedlander, Paul Keim

**Affiliations:** 1 Center for Microbial Genetics and Genomics, Northern Arizona University, Flagstaff, AZ 86011, USA; 2 Bioscience Division, Los Alamos National Laboratory, Los Alamos, NM 87545, USA; 3 Bacteriology Division, United States Army Medical Research Institute of Infectious Diseases, Fort Detrick, Frederick, MD 21702-5011, USA; 4 Pathogen Genomics Division, Translational Genomics Research Institute, Phoenix, AZ 85004, USA

## Abstract

**Background:**

An isolate originally labeled *Bacillus megaterium *CDC 684 was found to contain both pXO1 and pXO2, was non-hemolytic, sensitive to gamma-phage, and produced both the protective antigen and the poly-D-glutamic acid capsule. These phenotypes prompted Ezzell et al., (J. Clin. Microbiol. 28:223) to reclassify this isolate to *Bacillus anthracis *in 1990.

**Results:**

We demonstrate that despite these *B. anthracis *features, the isolate is severely attenuated in a guinea pig model. This prompted whole genome sequencing and closure. The comparative analysis of CDC 684 to other sequenced *B. anthracis *isolates and further analysis reveals: a) CDC 684 is a close relative of a virulent strain, Vollum A0488; b) CDC 684 defines a new *B. anthracis *lineage (at least 51 SNPs) that includes 15 other isolates; c) the genome of CDC 684 contains a large chromosomal inversion that spans 3.3 Mbp; d) this inversion has caused a displacement of the usual spatial orientation of the origin of replication *(ori) *to the termination of replication *(ter) *from 180° in wild-type *B. anthracis *to 120° in CDC 684 and e) this isolate also has altered growth kinetics in liquid media.

**Conclusions:**

We propose two alternative hypotheses explaining the attenuated phenotype of this isolate. Hypothesis 1 suggests that the skewed *ori/ter *relationship in CDC 684 has altered its DNA replication and/or transcriptome processes resulting in altered growth kinetics and virulence capacity. Hypothesis 2 suggests that one or more of the single nucleotide polymorphisms in CDC 684 has altered the expression of a regulatory element or other genes necessary for virulence.

## Background

Attenuated strains of *Bacillus anthracis *have played a major role in the development of vaccines and our understanding of anthrax. Early work by Pasteur and Greenfield [1,2] capitalized upon strains missing one of the mega-plasmids (pXO1), which resulted in attenuation. This enabled the development of the first bacterial disease to be prevented through the use of an attenuated live vaccine. This early work was improved by Sterne [3] through the development of an attenuated strain missing the second mega-plasmid (pXO2), but retaining the toxin producing genes on pXO1 as antigens for immune response. In recent years, avirulent strains have been subjected to extensive DNA sequencing to understand these plasmids, their virulence genes and to generate hypotheses for attenuation mechanisms [4-11]. Conversely *B. cereus *strains that have acquired the known *B. anthracis *mega-plasmids and anthrax-like virulence properties remain an enigma and are also worthy of further study to understand how this pathogen interacts with its host [12-14].

An isolate from the Centers for Disease Control (CDC) originally identified as *B. megaterium*, CDC 684/NRRL-349S/NRS 234 (herein called CDC 684), was being used as an avirulent outgroup control in experiments with *B. anthracis *[15]. However, this particular isolate shares key phenotypic traits with *B. anthracis *such as non-hemolytic on blood agar, production of protective antigen and the poly-D-glutamic acid capsule, and sensitivity to gamma bacteriophage. Because these features are all hallmark phenotypes for *B. anthracis*, Ezzell et al. [15] reclassified this isolate as *B. anthracis *despite the observation that CDC 684 did not react with monoclonal antibodies to a specific polysaccharide present in *B. anthracis*. Subsequent animal testing of this isolate showed it to be severely attenuated in guinea pigs, in contrast to wild-type *B. anthracis *(See results, **Attenuation of CDC 684**). However, the underlying mechanism behind this attenuated virulence phenotype remained unknown. The advent of massively parallel whole genome sequencing (WGS) provides an opportunity to examine the complete genetic component of CDC 684 for clues that might bear on this problem.

This report provides a description of the WGS, assembly and annotation of the *B. anthracis *CDC 684 isolate. We include analysis that: a) demonstrates that the genome of CDC 684 belongs to a specific *B. anthracis *clade; b) identifies 51 single nucleotide polymorphisms (SNP) that are unique to the genome of this isolate; c) describes the details of a large chromosomal inversion; d) demonstrates that CDC 684 has altered growth kinetics in culture and e) proposes two alternative and testable hypotheses that could explain the attenuated phenotype for CDC 684.

## Results

### Attenuation of CDC 684

The discovery that CDC 684 was not a *B. megaterium *strain but was rather *B. anthracis*, based on shared phenotypic features, prompted the use of the guinea pig model to determine its virulence. In a pilot experiment, groups of four guinea pigs injected i.m. with CDC 684 spores at doses of 114, 1,145, and 11,450 cfu/mL survived. These groups were then injected four days later with 1.29 × 10^5^, 1.29 × 10^6 ^and 1.29 × 10^7 ^cfu/mL, respectively, and again all survived. By comparison these identical spore preparation and treatment conditions produced LD_50 _values for the virulent Ames and Vollum-1B strains of 175 and 306 spores respectively in the guinea pig model [16,17].

This lack of lethality indicated that CDC 684 is significantly attenuated. In a second experiment to confirm attenuation, 10 guinea pigs injected i.m. with 1 × 10^8 ^cfu/mL CDC 684 spores all survived. These results confirm that CDC 684 is highly attenuated with an LD_50 _of >1 × 10^8 ^spores in the guinea pig model.

### WGS of CDC 684

The CDC 684 genome has been recently sequenced and assembled to closure at Los Alamos National Laboratory/J. Craig Venter Institute and is available on the NCBI Genome database [GenBank: CP001215.1]. The chromosome is 5,230,115 bp, pXO1 [GenBank: CP001216] is 181,773 bp and pXO2 [GenBank: CP001214] is 94,875 bp.

### Phylogenetic placement of CDC 684

The use of comparative WGS defined an extremely conserved and accurate phylogenetic SNP tree for *B. anthracis *based on the analysis of 1,000 SNPs in 26 diverse isolates [18]. This analysis resulted in the hypothesis that only a few selected SNPs at key positions along five branches were needed to accurately place all *B. anthracis *isolates into one of 12 sub-clades. This notion was shown to be accurate when 13 canSNPs were subsequently used to accurately place more than 1,000 *B. anthracis *isolates into one of these 12 sub-clades [19]. *In silico *canSNP typing showed that CDC 684 falls along the lineage created by *B. anthracis *Vollum (A0488; [GenBank: ABJC00000000]). This sequenced Vollum strain is presumed to be a close relative of the British isolate that was tested as a biological weapon on Gruinard Island, Scotland, in the 1940s [20].

The close phylogenetic relationship between CDC 684 and Vollum demonstrates that CDC 684 belongs to a highly virulent *B. anthracis *lineage. We were therefore interested in further determining the degree of relatedness between Vollum and CDC 684, given the marked differences in virulence between these two strains. An initial comparative *in silico *analysis of Ames Ancestor [GenBank: AE017334], CDC 684 and Vollum WGS uncovered ~ 390 SNP differences distinct from Ames Ancestor but common (i.e., derived) in both the CDC 684 and Vollum genomes. These results are consistent with other whole genome SNP comparisons of 128 *B. anthracis *isolates that suggest that the SNP genetic distance between Ames and Vollum is approximately 400 SNPs [Pearson, Schupp, Ravel and Keim, unpublished data].

**Figure 1:**
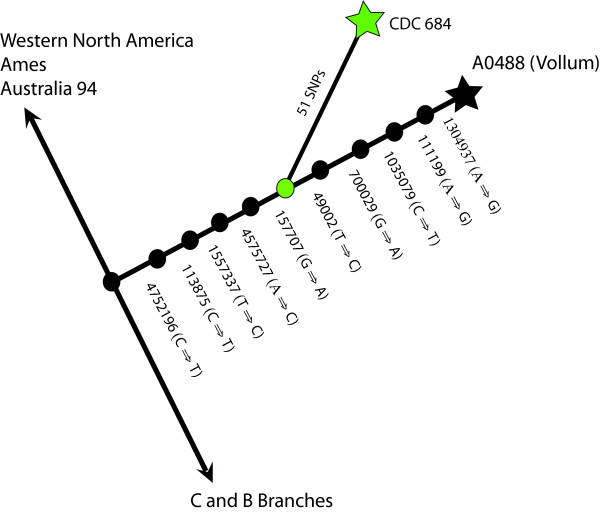
**Location of CDC 684 on the *B. anthracis *phylogenetic tree**. Genotypic analysis of 30 SNPs in ~ 100 isolates from the original Vollum node (Chung, Pearson and Keim, unpublished data) created 10 new collapsed branch points (nodes) along the Vollum sub-lineage (black circles). The positions of 10 new canSNPs are designated by the ancestral to derived SNP type in the Ames Ancestor and A0488 (Vollum) genomes respectively. The CDC 684 isolate creates a new branch (51 SNPs in length) midway along the Vollum branch and shares this node with 15 isolates that were obtained from a collection from the CDC [22].

Preliminary analysis of 30 SNPs that phylogenetically reside along a terminal position on the Vollum branch indicated that there were at least 10 new nodes along this branch, of which >100 Vollum-like isolates reside [Chung, Pearson and Keim, unpublished data]. *In silico *analysis of 10 new canSNPs along this branch indicated that CDC 684 was not in the terminal node created by the sequenced Vollum strain, but rather was located in a node midway between the sequenced strain and a branch point defined by the initial analysis of 100 Vollum-like strains, Figure 1.

### CDC specific SNPs

This analysis also demonstrated that CDC 684 possessed 51 SNPs that appeared to be unique to this isolate. There were 15 isolates that shared the Vollum branch node with CDC 684. These isolates were predominantly recovered by the Centers for Disease Control during the 1950s and 1960s. While the incidence of lethal anthrax infections in the United States had been greatly reduced during the 20^th ^century [21], it can be assumed that the majority of the CDC isolates labeled as *B. anthracis *would have come from sources containing virulent strains such as imported hides and/or animal deaths [22].

Table 1 lists 27 non-synonymous chromosomal SNPs from 51 total that are unique to CDC 684 in comparison to the Vollum (A0488) strain. There are no obvious *B. cereus *or *B. anthracis *virulence factors on this list but the role for each of these proteins in CDC 684 may also be compromised by the large inversion event. It also needs to be reiterated that while these SNPs are unique in their relationship to the Vollum strain their status in 15 other un-sequenced isolates who shared the node along the Vollum branch are still undetermined. It is likely that most of these SNPs will be shared (i.e., no differences) with these 15 presumably virulent *B. anthracis *isolates.

**Table 1:** CDC 684 specific non-synonymous SNPs indicating chromosomal positions, gene products and amino acid changes

Gene	Position	Product	*A*-*aa*	*D*-*aa*
GBAA0382	47140	ABC transporter, substrate binding	W	*
GBAA0414	435861	Hypothetical protein	I	V
GBAA0492	488260	Amino Acid permease family	A	V
GBAA0715	734957	ABC phosphate binding protein	Q	*
GBAA0925	935719	Putative lipoprotein	E	G
GBAA1301	1248597	PAP 2 family protein	S	N
GBAA1858	1742404	Major facilitator transporter	V	G
GBAA2173	2023504	Conserved hypothetical protein	G	D
GBAA2372	2212248	Non-ribosomal peptide synthetase	V	A
GBAA2649	2470526	Putative permease	M	V
GBAA2936	2704543	Putative membrane protein	P	S
GBAA4328	3953215	Conserved hypothetical protein	A	T
GBAA4328	3953216	Conserved hypothetical protein	A	V
GBAA4353	3973954	Acetylglutamate kinase	G	R
GBAA4388	4002699	Phosphate butyryl transferase	A	V
GBAA4408	4021751	Acetyl-CoA carboxylase	Q	*
GBAA4430	4037923	Hypothetical protein	A	T
GBAA4515	4109405	RNA polymerase sigma-43	K	R
GBAA4516	4110631	DNA primase	P	L
GBAA4521	4113151	CBS domain protein	A	V
GBAA4733	4305747	ABC-transporter, ATP-binding	G	R
GBAA4890	4443754	Thiol peroxidase	N	S
GBAA5207	4726424	Conserved hypothetical protein	T	M
GBAA5377	4870264	SpoVA family protein	I	V
GBAA5530	5021797	Conserved hypothetical protein	E	K
GBAA5678	5165710	ABC transporter, ATP-binding	A	T
GBAA5703	5193199	RNA helicase, DEAD/DEAH box	A	V

### Sequence variations between the virulence plasmids

The simplest explanation for the attenuated phenotype for CDC684 would be the mutation of one or more of the virulence factors encoded on the pXO1 or pXO2 plasmids that altered expression or function. These virulence factors include the toxin gene complex on pXO1 (comprising genes encoding for protective antigen, edema factor, and lethal factor), the poly-D-glutamyl capsule gene complex on pXO2 (encoded by *capA, capB, capC *and *acpA*), and trans-acting transcription regulators on both plasmids [23]. However, *in silico *comparison of the completed sequences of the pXO1 and pXO2 plasmids from the CDC 684 strain to those of the Ames Ancestor and Vollum strains showed that all of the known virulence factors were intact. There was a single non-synonymous SNP found in pXO1 GBAA_pXO1_0019, a large gene of unknown function. Collectively we observed no putative functional differences in the plasmid-encoded virulence factors between CDC 684 and its closest relative, Vollum, which is a fully virulent strain [24].

### Large chromosomal inversion in CDC 684

**Figure 2:**
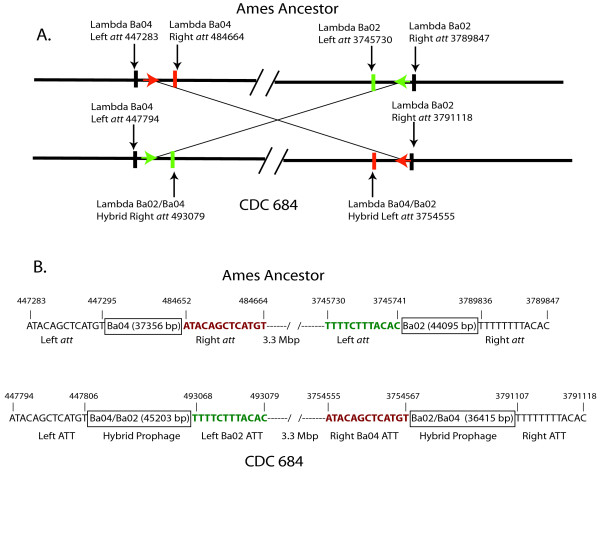
**The chromosomal inversion in CDC 684**. Panel A: The *att *sites in two lambda-like prophages, Ba04 and Ba02. The *att *sites are illustrated as vertical bars and their positions define the insertion site for each prophage. The black vertical bars indicate the position of the external flanking edge of the *att *sites in each prophage. The black *att *sites are in the same relative positions in both the CDC 684 and Ames Ancestor genomes. However, the red and green *att *sites (bars) highlight the positions in the prophages that are inverted in the CDC 684 genome. The red and green arrows indicate putative sites where a homologous exchange caused the 3.3 Mbp inversion. Panel B describes the unique *att *sites and defines the size of each of the prophages. This panel also illustrates the translocation of the internal (red and green) *att *sites in CDC 684 to equivalent positions within the sister prophages, which are 3.3 Mbp apart. The new *att *sites for both Lambda Ba04 and Lambda Ba02 indicate that these two prophages in CDC 684 are now hybrid prophages containing unique 3' elements.

The most striking feature of CDC 684 genome is a massive inversion that reverses the orientation of 3.3 Mbp of the chromosome relative to the replication origin. The dimensions of the inversion have been graphically illustrated in a recent review of *Bacillus anthracis *genome variation [25]. This earlier report used Artemis software http://www.sanger.ac.uk/resources/software/artemis/ to illustrate the alignment and conserved gene order of four finished and closed genomes (*B. anthracis *Ames, *B. anthracis *Australia 94, *B. anthracis *CDC 684, and *B. thuringiensis *Al Hakam). While the fine-scale gene order in CDC 684 is precisely maintained as in the Ames chromosome, the large rearrangement has caused an inversion of a 3.3 Mbp region between the basepair coordinates 454 Kbp and 3,783 Kbp in the Ames Ancestor chromosome (see Figure 2).

The inversion appears to have been caused by an internal recombination event between homologous regions within two lysogenic lambda-like prophages (LambdaBa04 and LambdaBa02), which are found in all *B. anthracis *genomes [26,27]. The inversion can best be visualized at the molecular level by examining the orientation of the *att *(attachment) sites that flank the ends of these phages (Figure 2). Lysogenic bacteriophages possess cohesive ends *(att)*, usually 12-13 bp repeats, which serve as both excision points and "sticky ends" that enable the phage to cirularize as it enters a lytic life cycle [28]. At first glance it seemed likely that the inversion may involve the *att *sites in these Lambda like prophages and that the exchange may have involved a site-specific recombination. But the two *att *sites were unique to each other, i.e., Lambda Ba04 and Ba02 contain distinct *att *sites (Figure 2B) that allow them to be distinguished from each other (Ba04, ATACAGCTCATGT and Ba02, TTTT(C/T)TTTACAC). In Ames Ancestor, pairs of these two distinct *att *sites define both the size (Ba04 = 37.3 kb; Ba02 = 44.0 kb) and boundaries of each prophage. In CDC 684 (Figure 2A), the external *att *sites (represented by black bars) are in relatively identical chromosomal positions to those in the Ames Ancestor. However, the internal *att *sites (represented by green and red bars) have dramatically exchanged positions between these genomes. In CDC 684, the right *att *site (red bar) for LambdaBa04 has moved to the left *att *position of Lambda Ba02, and likewise the left *att *site for Lambda Ba02 (green bar) has moved to the position occupied by right *att *site in Lambda Ba04. The net effect of this exchange is the creation of new hybrid prophages in CDC 684 (Figure 2B). These observations indicate that the large inversion event did not involve site-directed recombination but rather a homologous recombination event in the interior of both prophages.

### Molecular detection of the inversion in other *B. anthracis *strains

**Figure 3:**
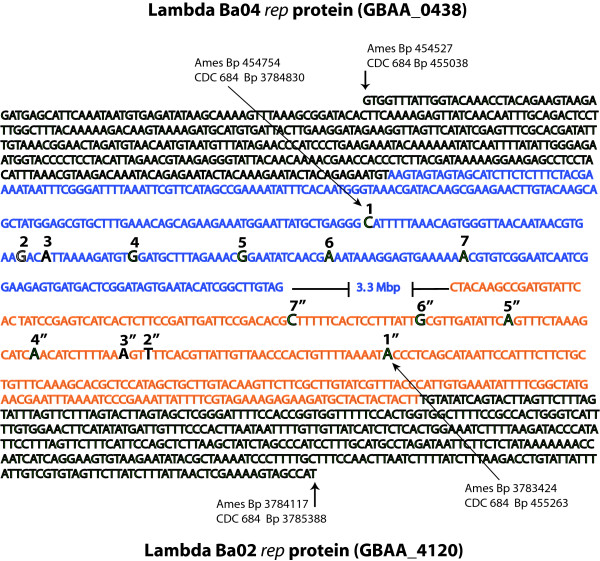
**Site of the CDC 684 inversion**. This figure contains the entire sequences for the Lambda Ba04 and Lambda Ba02 *rep *genes. The 5' nucleotides in black represent regions unique to the prophage *rep *genes and are in the same directionality in the CDC 684 and Ames Ancestor genomes. The blue and orange nucleotides represent the 3' ends that are homologous to both *rep *genes, and likely represent the site of recombination that resulted in the 3.3 Mbp inversion in CDC 684. Seven SNPs that define the prophage 3' ends are in larger font, and are labeled 1-7 or 7"-1" for Lambda Ba04 or Lambda Ba02, respectively. In CDC 684, the allele states for these prophages have switched chromosomal positions relative to Ames Ancestor.

A PCR approach was designed to detect the inversion sites in CDC 684 as a method that could test for the presence of the inversion in other isolates. Because of its size, the inversion is readily visible in the closed genome, but the molecular nature of the inversion is dependent on the proper alignment of two short regions (i.e. 165 bp) during the assembly of this genome. As illustrated in Figure 3, the 5' end of each of the *rep *sequences are distinct from each another and their positions are fixed at approximately the same positions in both genomes. However, the 3' end of the *rep *genes are highly homologous, with scattered SNPs the only distinguishing feature between these paralogs.

Due to constraints on PCR amplicon size we used mismatch amplification mutation assays (MAMA, [29]) to discriminate between the right and left ends of the large inversion in CDC 684 and Ames Ancestor. The rationale was to demonstrate the different ends of the inverted 3.3 Mbp fragment in CDC 684 by use of real time PCR assays. The MAMA system was designed to take advantage of polymorphic differences that characterize the left and right SNP signatures within the *rep *Lambda-like protein sequences relative to the Ames Ancestor genome. Both the left and right assay systems have common primers (CP, Table 2 and Methods) that are fixed because they are external to the 3.3 Mbp inversion site. The internal primers are nearly identical but they target mismatches at specific SNP sites; G on the left site and A on the right site of the Ames genome. These same internal primers (e.g., CP-Left-Inv-F and Left-Inv-R, Table 2) cannot amplify the same 400 and 500 bp products in CDC 684 because they are separated by 3.3 Mbp. But the reciprocal pairings of the internal primers do amplify products from CDC 684.

**Table 2:** MAMA assays used to detect the CDC 684 chromosomal inversion

*Left Inversion Primers*	Polymorphism	Assay Targets
**CP Left-inv-F + Right-inv-F**	A	CDC 684
**CP Left-inv-F + Left-inv-R**	G	All other *B. anthracis*

***Right Inversion Primers***	**Polymorphism**	**Assay Targets**

**Left-inv-R + CP Right-inv-R**	G	CDC 684
**Right-inv-F + CP Right-inv-R**	A	All other *B. anthracis*

Where CP = Common Primer; inv = Inversion; F = Forward; R = reverse

These MAMA were used to analyze several isolates within the Vollum branch. In addition, the SNPs flanking the inversion were compared to *in silico *analysis of other *B. anthracis *WGS to determine the configuration of this 3.3 Mbp region in other non-Vollum strains. Table 3 illustrates that only the CDC 684 isolate possessed the inverted genotype from among 17 genomes examined, indicating the inversion is not common in *B. anthracis*.

**Table 3:** Status of the Large Inversion Site by PCR or *in silico *analysis **of 18 ***B. anthracis *genomes

Isolate	Lineage	Assay	Orientation
A0488	Vollum	Real-time PCR	Ames-like
A1136	Vollum	Real-time PCR	Ames-like
A1093	Vollum	Real-time PCR	Ames-like
A1094	Vollum	Real-time PCR	Ames-like
A0363	Vollum	Real-time PCR	Ames-like
A0474	Vollum	Real-time PCR	Ames-like
CDC 684	Vollum	Real-time PCR	CDC 684
A0493	W.N.A.	*In silico*	Ames-like
A0442	Kruger B	*In silico*	Ames-like
A0402	CNEVA	*In silico*	Ames-like
Tsiankovskii	A.Br.008/009	*In silico*	Ames-like
A0174	W.N.A.	*In silico*	Ames-like
A0465	CNEVA	*In silico*	Ames-like
A0389	A.Br.001/002	*In silico*	Ames-like
A0193	W.N.A.	*In silico*	Ames-like
A2012	Ames	*In silico*	Ames-like
A0248	Aust 94	*In silico*	Ames-like
A1055	C-Branch	*In silico*	Ames-like

### Defining the *dif *site in *B. anthracis*

In *E. coli *the large *ter *region has been found to contain a specific substrate sequence, *dif *(for Deletion Induced Filamentation), which is used by two recombinases, XerC and XerD, to resolve chromosomal multi-mers and to allow daughter chromosomes to segregate before cell division [30,31]. It has been proposed that the *dif *site (a short palindromic sequence) is in fact a more likely site of termination than any specific *ter *sites for both the *E. coli *and *B. subtilis *chromosomes [32]. From the perspective of the CDC 684 genome, the *dif *sites in both γ-proteobacteria and Firmicutes appear to have an extremely close association with the maximum GC-skew in those genomes that have been analyzed [32,33].

**Figure 4:**
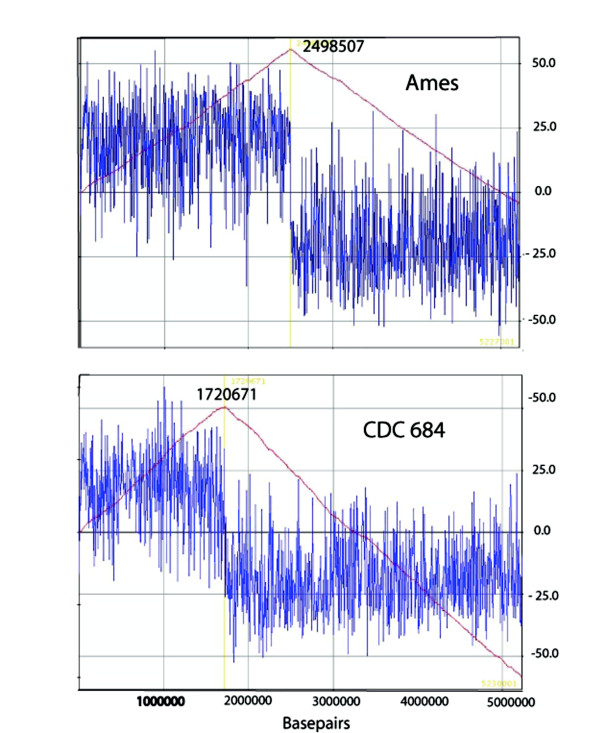
**GC Skew Plot for *B. anthracis *Ames and *B. anthracis *CDC 684**. GenSkew http://genskew.csb.univie.ac.at/, was used to compute the cumulative GC skew for these two complete genomes. Note that the position denoting the maximum skew for CDC 684 has shifted dramtically in comparison to the Ames Ancestor genome. This suggests that the terminus of replication for CDC 684 may be in an altered positon.

*Dif *sites have been defined in both *B. subtilis *[34] and a member of *B. cereus *sub-group [32]. A cursory survey of the palindrome from the *B. subtilis *and *B. cereus dif *site (AATATATATT) in the Ames Ancestor identified a 28-bp palindromic sequence [32] that is located at nearly the precise genomic site of the cumulative GC-skew. This sequence is conserved and positioned at the cumulative ~ 180° GC-skew position of every complete whole genome sequence in all of the GenBank entries for the *B. cereus *sub-group (Table 4). The one exception is the genome of CDC 684 where the conserved *dif*-like sequence and the GC-skew are oriented at ~ 120° in relationship to the origin of replication (Figure 4, Table 4).

**Table 4:** Chromosomal locations of GC-skew, *dif *sites and their relative orientation in relationship to the Origin of Replication in complete genomes.

Isolate	Genome	GC Skew	*dif *Position	*Orientation
Bc biovar Ba CI	5196054	2514865	2516079	174°
Bc 03BB102	5269628	2587080	2592759	177°
BcQ1	5214195	2507935	2510631	171°
BcAH187	5269030	2560736	2564400	174°
Bc AH820	5301683	2566169	2575244	174°
Bc B4264	5419036	2617378	2620353	174°
Bc E33L	5300915	2570501	2571014	174°
Bc ATCC 14579	5411809	2673035	2681358	178°
Bc ATCC 10987	5224283	2585881	2590339	178°
Bc G9842	5387334	2591148	2591275	173°
Ba Ames Ances.	5227419	2498507	2507867	172°
CDC 684	5230115	1720671	1732304	119°
Bt 97-27	5237682	2529472	2560322	173°
Bt Al Hakam	5257091	2591702	2593007	177°
Bt BMB171	5330088	2601041	2608011	176°

### Growth Kinetics of CDC 684 versus wild type *B. anthracis*

The significant difference in the spatial orientation of the *ori *site and *dif*/GC skew sites in CDC 684 suggests that there could be an alteration in how the bi-directional replication of chromosome would proceed because of the unequal distances the opposite leading strands would need to travel. Because accumulated evidence indicates that genomes like those of *E. coli *and *Bacillus sp *do not tolerate significant changes between the spatial orientation of the *ori *and *ter *sites, we designed a growth experiment to compare the growth kinetics of CDC 684 to those of three wild type *B. anthracis *strains.

**Figure 5:**
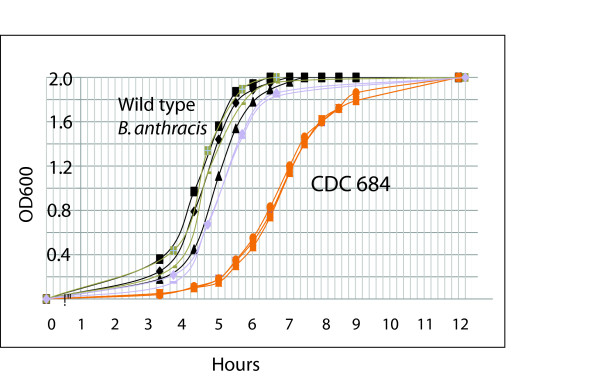
**The kinetics of growth of wild type and CDC 684 *B. anthracis *isolates on LB broth**. Growth curves for four strains of *Bacillus anthracis*: Ames, Ba_A0361 (a B branch isolate), Vollum and CDC 684 were obtained for these isolates growing on LB broth at 37°C. These cultures were grown in duplicate (Ames, BaA0361) or triplicate (Vollum, CDC 684) with growth measured indirectly by OD_600_.

Growth curves for four strains of *Bacillus anthracis*: Ames, Ba_A0361 (a B branch isolate), Vollum and CDC 684 were grown in LB broth at 37°C (Figure 5). These cultures were grown in duplicate (Ames, BaA0361) or triplicate (Vollum, CDC 684) with growth measured by OD_600_. The strains represent two major phylogenetic groups of *B. anthracis*. Note the relatively consistent growth curves for the three wild type isolates: Ames, Ba A0361 and Vollum, the closest relative to CDC 684. Two obvious differences between the CDC 684 and Vollum growth curves is a longer lag phase and slower mid log growth rate in CDC 684. These differences were noted despite careful efforts to exactly match inoculum sizes using direct plating viability counts. An extended lag phase would suggest that CDC 684 takes longer to adapt to the inoculum transfer process and/or to conditions necessary for growth and cell division. The slower mid log growth rate (~55 min in Vollum and ~80 min in CDC684) in this experiment suggests that even after revival from lag phase that CDC 684 has a cellular limitation to growth that does not exist in the wild type strains. These results provide a growth parameter that implies that the spatial change in the orientation of the origin of replication and the termination site in CDC 684 may have altered the growth of this isolate.

## Discussion

By phylogenetic, molecular and clinical criteria, CDC 684 is a *B. anthracis *and its attenuated phenotype must be due to differences within its genome relative to those of other closely related *B. anthracis *strains. The marked degree of attenuation of CDC 684, (with an LD50 of >1 × 10^8 ^spores by the i.m. route in the guinea pig) compares with LD50 values of 175 and 306 spores reported for the virulent Ames and Vollum-1B strains [16,17]. It therefore renders a comparative genomics approach highly informative and suggests that either subtle SNP differences and/or a dramatic and massive inversion within this chromosome are responsible for the attenuation.

Whole genome sequencing and comparative analysis indicates that there are 51 chromosomal and < 6 plasmid SNP that are unique to CDC 684 in a comparison to Vollum. The possibility that one or more of these rare SNPs may have an important role in the attenuation of CDC 684 remains a viable option. These data have defined a new CDC 684 lineage emanating from the original Vollum branch, Figure 1. Twenty-seven of these SNPs would be translated into non-synonymous mutations in putative gene functions. None of these SNPs, however, are in genes considered to be virulence factors found in opportunistic *B. cereus *pathogens that include a variety of hemolysins, non-hemolytic enterotoxins, monomeric entertoxins and phospholipases [35]. The remaining 23 SNPs include 11 synonymous SNPs, 7 SNPs in pseudogenes, and 5 intra-genic SNPs. Only one of these intra-genic SNPs is located in a region within a promoter region (-7 bp) in a L-serine dehydratase gene (GBAA_4361).

What has not been excluded from this new lineage are 15 *B. anthracis *isolates that currently share the nodal position between the CDC 684 and Vollum lineages (see Figure 1). A sequencing effort to identify CDC 684 specific SNP that are either shared or still unique among the presumably virulent 15 isolates would point to phenotype altering SNP. Any chromosomal and plasmid SNP that are still unique to CDC 684 would be candidates for having positions in genes or regulatory regions with roles that govern known or unknown functions that are necessary in a virulent organism. There is, as yet, no clear notion whether or how any of these SNPs could cause the dramatic change in the virulence or growth properties of CDC 684.

The role of the chromosome of *B. anthracis *in the overall etiology of the disease anthrax is still poorly understood. It is becoming evident that the regulatory functions of the virulent plasmids (pXO1 and pXO2) work in concert with certain chromosomal regulatory functions in a virulent organism, e.g. the regulation of the pXO1 *atxA *gene by chromosomal sigma factors or plasmid genes involved in a signal-transduction pathway that inhibits sporulation [36]. These and other recent studies [37] make it difficult to dismiss any of the CDC 684 non-synonymous mutations as candidates for a role in the attenuated phenotype without further analysis.

**An alternative hypothesis **to explain the attenuation of CDC 684 is a role for the large 3.3 Mbp inversion within its chromosome. While this inversion does not appear to have altered the fine-scale order of the individual genes, it has changed the orientation of the genes within the inversion with respect to the genes outside of the inversion. This change in the orientation has been illustrated by whole genome alignments [25] and by an analysis of the GC skewing and the location of *dif *sites of the CDC 684 genome and that of several *B. anthracis *and *B. cereus *sub-group isolates (Figure 4, Table 4). These analyses indicate that the spatial relationship between the origin of replication and the termination of replication in CDC 684 has been perturbed by the massive inversion. The comparative growth data (Figure 5) clearly supports the idea that chromosomal replication may be altered in CDC 684 by exhibiting an extended lag phase and a longer growth rate.

The longer DNA synthesis time needed to complete chromosomal replication may be sufficient, alone, to explain the slower cellular growth rate of CDC 684. In the asymmetrical CDC 684 chromosome, the longer leading strand distance is 3.783 Mbp vs. 2.615 Mbp for Vollum and all characterized wild type *B. anthracis *strains. This is a ~38% larger chromosomal distance to replicate and, assuming everything else remains constant, this will take that much longer to complete the entire chromosome. The mid log doubling time difference between the wild type strains (~80 min) and CDC 684 (~80 min) is ~45%. The similarity between the 38% long replication distance and 45% longer growth rate is striking. This observation suggests that the displaced *ter *region remains the site for replication termination and that the asymetrical longer leading strand replication distance in CDC 684 becomes limiting for growth *in vitro*.

Historical accounts suggest that there are strong tendencies to conserve the basic relationship between the position of the *ori *and *ter *sites in enteric bacteria [38]. Following the discovery of the *dif *sites and related specific recombinases, it was proposed that the topological relationship between the *ori *and the *ter/dif *site must be maintained at 0° and 180°, respectively, for normal chromosomal segregation to occur [31]. This was suggested because mutations in the Xer recombinase genes or the *dif *site or the displacement of the *dif *site to other regions of the chromosome had adverse effects on cell division.

More recently whole genome sequence comparisons between several distinct species also suggest that there is conservation in the spatial orientation between the *ori *and *ter *sites over broad groups of bacteria [39-41]. Dot plots of conserved DNA and protein sequences between pairs of species produce characteristic X-shaped patterns suggesting that large chromosomal rearrangements often revolve around and maintain the distances between the origin and the terminus.

This study illustrates a case where the naturally conserved 180° orientation of the *ori *and *ter *sites has been modified by a large chromosomal inversion in a strain of *B. anthracis*, CDC 684. We suggest that the consequence of the altered spatial relationship between the *ori *and *ter *sites from 180° to 120° has caused the change in growth kinetics of this isolate (Figure 5). We also suggest that this change appears to alter the length of time that CDC 684 takes to replicates its chromosome. Whether this change has also altered the virulent phenotype of this isolate is yet to be determined.

## Conclusions

We address two hypotheses that could have a role for the attenuated phenotype in *B. anthracis *CDC 684. The first is that a single chromosomal point mutation may have altered a function that is crucial to normal growth and virulence in *B. anthracis*.

Despite evidence for a wide array of chromosomal rearrangements in the *B. cereus *subgroup [42], BLAST searches conducted using the *dif *region of *Bacillus anthracis *and *B. cereus *also indicate a trend towards maintaining a nearly 180° spatial relationship between the *ori/dif *sites (171°- to 178°, Table 4). The second hypothesis, therefore, suggests that major alterations of this relationship are possibly not tolerated by *B. anthracis *when under natural ecological pressures. The existence of an isolate like CDC 684 demonstrates that a moderate skewing in the spatial relationship between the *ori */*dif *may be overcome in terms of sheer growth and survivorship in the laboratory. But we suggest that the potential biological consequences of altered DNA replication and/or DNA expression rendered by this change may have resulted in an altered phenotype for successful pathogenicity in a mammalian host. Both kinds of "genetic alterations" can be expected to be rare in *B. anthracis *since these organisms would not have a selective advantage in a natural environment and would be difficult to find.

## Methods

### Whole genome sequencing and assembly

The genome of *B. anthracis *CDC 684: Chromosome [GenBank: CP001215.1]. pXO1 [GenBank: CP001216] and pXO2 [GenBank: CP001214] was sequenced at the Joint Genome Institute (JGI)/J. Craig Venter Institute using a combination of 3 kb and 8 kb DNA libraries. All general aspects of library construction and sequencing performed at the JGI can be found at http://www.jgi.doe.gov/. Draft assemblies were based on 59,691 total reads. The Phred/Phrap/Consed software package http://www.phrap.com was used for sequence assembly and quality assessment [43,44]. After the shotgun stage, reads were assembled with parallel Phrap (High Performance Software, LLC). Possible mis-assemblies were corrected with Dupfinisher [45] or transposon bombing of bridging clones (Epicentre Biotechnologies, Madison, WI). Gaps between contigs were closed by editing in Consed and by custom primer walking (Roche Applied Science, Indianapolis, IN). A total of 1955 additional custom PCRs were necessary to close gaps and to raise the quality of the finished sequence. The completed genome sequence of *B. anthracis *str. CDC 684 contains 62,606 reads, achieving an average of 10-fold sequence coverage per base with an error rate of < 10^-6^.

### Experimental animals and spore challenges

Spores were prepared from *B. anthracis *CDC 684 as previously described [16] and female Hartley guinea pigs (660 g) were challenged intramuscularly (i.m.) with various spore concentrations (see 'Results') at USAMRIID as previously described [16,46]. Research was conducted in compliance with the Animal Welfare Act and other federal statutes and regulations relating to experiments involving animals and adheres to principles stated in the *Guide for the Care and Use of Laboratory Animals *(National Research Council. 1996. Guide for the care and use of laboratory animals National Academy Press, Washington, DC.). The facility where this research was conducted is fully accredited by the Association for Assessment and Accreditation of Laboratory Animal Care International.

### Canonical SNP (canSNP) Analysis

The thirteen canSNP alleles and the specific assays for each have been described previously [19]. TaqMan™ Minor Groove Binding (MGB) allelic discrimination assays were used to determine the precise canSNP grouping for every isolate used in this study [19,47].

### SYBR MAMA Assays

Additional SNP genotyping was conducted using the Mismatch Amplification Mutation Assay [MAMA] [29], which is based on allele-specific PCR kinetics [48], enhanced by penultimate mismatch primer design [29,49]. The MAMA approach was also used to distinguish the inverted 3.3 Mbp segment of CDC 684 from all other *B. anthracis *strains. MAMA assays were designed for both the 5' (left) and 3' (right) ends of the inversion; i.e., two sets of primer products separated by 3.3 Mbp. The sequences flanking the 3.3 Mbp inverted region were unique and common to both CDC 684 and the Ames genomes and were defined as Common Primers (CP). But the internal primers targeted nearly identical sequences and therefore used primers designed around mismatches that could distinguish and generate 400 and 500 bp PCR products. The primers were as follows (5' to 3'): Left-inv-R (TAAAGCATCCACATCTTTTAATGgC), Right-inv-F (TTTCTAAAGCATCAACATCTTTTAAAGgT), and CP-Left-inv-F (GCATGTGATTACTTGAAGGATAGAAGG) were used to characterize the left inversion, and Left-inv-R, Right-inv-F and CP-Right-inv-R (5'- AGATTTCCAGTGAGAGATGATAACAACA) targeted the right inversion. Underlined nucleotides overlap the SNP; nucleotides in lowercase represent deliberate penultimate mismatches. The two consensus primers contained no SNPs or incorporated mismatches. Expected inversion genotypes using these primers are listed in Table 2 in the Results section and an example of this assay system is illustrated in Additional File 1.

The MAMA assay system was also used to type 10 new canSNP sites that further define the Vollum lineage of *B. anthracis*. The primers for these sites are shown in Additional File 2 as a Table.

Each inversion SYBR MAMA reaction comprised 1X SYBR Green Master Mix (Applied Biosystems, Foster City, CA), 0.1 uM MAMA primer, 0.2 uM consensus primer, 0.08 U Platinum *Taq *polymerase (Invitrogen, Carlsbad, CA) and molecular grade H_2_O to 9 uL. One uL of genomic DNA was added to each well to a final volume of 10 uL. Reactions were carried out in 384-well optical plates (Applied Biosystems) on an ABI Prism 7900 HT real-time instrument (Applied Biosystems) using the following thermocycling parameters: 2 min at 50°C, 10 min at 95°C, followed by 50 cycles of 15 s at 95°C and 1 min at 60°C. PCR products were subject to post-amplification dissociation (15 sec at 95°C, 15 sec at 60°C, 15 sec at 95°C) to confirm product specificity.

Additional File 1 provides an example of real time PCR profiles for the left inversion region using a fixed Common Primer (CP) that is located outside of the left boundary of the 3.3 Mbp inversion site in both CDC 684 and the Ames genomes. This figure demonstrates real time PCR kinetics for the detection of amplicons for the left boundary of the inversion site in both CDC 684 and the Ames Ancestor Genome using primer combinations described in Table 2.

### GC Skew Analysis

A free software program, GenSkew http://genskew.csb.univie.ac.at/, was used to compute the cumulative skew for 15 complete WGS of *B. anthracis, B. cereus *and *B. thuringiensis*. These WGS data were downloaded from GenBank: http://www.ncbi.nlm.nih.gov/genbank/.

### Growth Data

Stocks of *B. anthracis *Ames, *B. anthracis *Vollum (A0488), *B anthracis *A0361 (a B branch isolate), and *B. anthracis *CDC 684 were subcultured and grown for ~19 hours on LB agar. These cells were harvested and normalized to OD_600 _densities that correspond to 10^5 ^cfu/mL based on viable count estimates from previous experiments for each isolate. These measurements were used to precisely add 10^5 ^cfu inoculums to create 3 ml culture tubes for each isolate. These cultures were grown at 37° C and OD_600 _measurements were determined on a CO800 Spectrophotometer.

## Authors' contributions

All authors have read and approved the final version of the manuscript. RTO contributed to the interpretation of data and in generating the original draft of the manuscript. EPP made substantial contributions in design of experiments, acquistion and interpretation of data and in the critical editing of the manuscript. SRW made substantial contributions in acquisition and interpretation of data. JMG made substantial contributions in data acquired using bioinformatic tools and includes the visualization of the inversion. WKC was responsible for the generation of canSNP data and analysis. TP made substantial contributions in the development of specific markers and in phylogenetic analysis of CDC 684 and its relationship to the world-wide distribution of *B. anthracis*. GX was an important part of the Genome Sequencing team from Los Alamos who generated, completed and annotated the CDC 684 WGS. CM was an important part of the Genome Sequencing team from Los Alamos who generated, completed and annotated the CDC 684 WGS. KKH provided the impetus to acquire the CDC 684 isolate, confirmed the *B. anthracis *like genotype, initiated the eventual WGS of this isolate and provided a critical review and edit of the draft manuscript. JC provided the genome announcement and initial annotation of CDC 684 WGS as part of the Genome Sequencing team from Los Alamos. BEI conducted the animal spore challenges in Guinea pigs that demonstrated the attenuated phenotype of CDC 684. JMS provided comparative whole genome analysis of CDC 684 against other whole genome sequences and the identification of SNPs that are unique to the CDC 684 genome. SMB provided comparative whole genome analysis of CDC 684 against other whole genome sequences and the identification of SNPs that are unique to the CDC 684 genome. AF provided the animal spore challenge data and the interpretation of this data as well as a critical review of the initial draft of this manuscript. PK provided the inspiration and the major intellectual content for this work, was the first to describe the large inversion event and provided the first and final critical reviews of this document.

## Supplementary Material

Additional file 1 **Figure S1: MAMA real time PCR**. Example of MAMA Real Time PCR to Differentiate between a 3.3 Mbp inverted configuration in CDC 684 and the wild type configuration in *Bacillus anthracis*. The primers depicted in this figure were designed to detect the left configuration for the wild-type and/or the inverted sequence for the CDC 684 genome. The fixed common primer, CP Left-inv-F was paired with both Right-inv-F and the Left inv-R primers. While the wild type Ames Ancestor can be amplified by the two LEFT inversion primers (Left inv F and Left inv R); the left inversion site in CDC 684 can only be amplified by the CP left-inv-F and Right inv-F because the Right inversion forward site normally situated 3.3 Mbp distal, has relocated to within 450 bp of the CP left-inv-F primer. Note that these two panels represent samples with the same three primers and that the positive allele will amplify orders of magnitudes faster than the negative allele. The delta CT (cross-over threshold) values are the differences between a specific linear portion of the target sequence (in cycles) versus the number of cycles to obtain the same (threshold) signal in the non-target sequence (e.g....the delta CT for CDC 684 vs Ames is 13.4. Click here for file

Additional file 2 **Table S1: The SYBR MAMA primer sets for 10 Vollum lineage canSNP Assays**. Click here for file
