# Supplementary material for: An attenuated strain of Bacillus anthracis (CDC 684) has a large chromosomal inversion and altered growth kinetics
Source: BMC Genomics. 2011 Sep 30;12:477. doi: 10.1186/1471-2164-12-477 (PMC3210476; doi:10.1186/1471-2164-12-477)
Supplement: Additional file 2 — Table S1: The SYBR MAMA primer sets for 10 Vollum lineage canSNP Assays. [file 1471-2164-12-477-S2.DOCX]

| Ames Ancestor Position | Forward Ancestor Primer 5’-3’ | Forward Derived Primer 5’-3’ | Consensus Reverse Primer 5’-3’ |
| --- | --- | --- | --- |
| 1304937 | CTATTGTATCAAAATTTGAGTTATCAGGAAAA | CTATTGTATCAAAATTTGAGTTATCAGGAATG | GCTCTGCCACGCTATTAAAATGAA |
| 111199 | agtaaggcatatcttcttctggtaaaGT | ctggtaagtaaggcatatcttcttctggtaaaTC | gaaagaaacacttatcgcagttgaaaaAA |
| 1035079 | CGTTGGGCTTGATCCGTTACC | TCGTTGGGCTTGATCCGTTATT | TTCGCTCTTACCTCGCCTTGATG |
| 700029 | AAGTAAATCAAGATTGAACACGTTAAG | AAGTAAATCAAGATTGAACACGTTACA | acgcacttgacttgtcttc |
| 49002 | attttcatactctcacctcaaatcacgaaCA | ctaattttcatactctcacctcaaatcacgaaTG | cagatgctgtattaatgagtggaagtgg |
| 157707 | AGAAAAGGAACGGATACAAACTAGCAG | AAAAGGAACGGATACAAACTAGCCA | ACACATGTTTAGTCGCCGTTAC |
| 4575727 | CGCAAGAATTGTTCTAAATCGATTCTC | CGCAAGAATTGTTCTAAATCGATTCTC | GTATATGAACTTGGCGCTGATGAAGTATG |
| 1557337 | GCAATTGGTAATCCAGCATACTTAACCT | CAATTGGTAATCCAGCATACTTAACGC | AACTAACTCCTCATGATTTTGGTGCTATT |
| 113875 | ACGTTTCAAAATTACAGAAACGTCTCAC | CGTTTCAAAATTACAGAAACGTCTCCT | TACTTTTGCTTGTGCTTCGTGG |
| 4752196 | cgaaagaaacacttatcgcagttgaaaaTG | gaaagaaacacttatcgcagttgaaaaAA | taaaggtattaaactgtataacgctcatcttctaa |

### Additional file 2 – Table: The SYBR MAMA primer sets for 10 Vollum lineage canSNP Assays
